# mTORC2 takes the longevity stAGE

**DOI:** 10.18632/oncotarget.2457

**Published:** 2014-09-05

**Authors:** Dudley W. Lamming

**Affiliations:** Department of Medicine, University of Wisconsin-Madison and William S. Middleton Memorial Veterans Hospital, Madison, WI

Rapamycin, a FDA-approved pharmaceutical, has received significant attention as a possible anti-aging agent since the groundbreaking discovery in 2009 that rapamycin treatment significantly extends the lifespan of mice [1]. Excitingly, rapamycin not only extends lifespan, it also extends healthspan in rodent models, preventing and delaying the onset of age-related diseases, including cancer and Alzheimer's disease (reviewed in [2]). Rapamycin rejuvenates the aging mouse heart, and ameliorates age-related cognitive decline in multiple mouse models. Unfortunately, the therapeutic potential of rapamycin for age-related diseases has not been fully realized due to significant fears about its side effects. These include immunosuppression and metabolic side effects such as dyslipidemia, the development of glucose intolerance and hepatic insulin resistance. Whether or not rapamycin-induced hepatic insulin resistance is a negative side effect, or instead reflects a starvation-induced protective state that promotes longevity, is a subject of ongoing research [3]. Rapamycin in mice has sexually dimorphic effects, with a more beneficial effect in females than males [4], which may further complicate the translation of rapamycin therapy to humans.

In order to better understand the mechanisms underlying both the beneficial and deleterious effects of rapamycin, we focused our attention on the mechanism by rapamycin affects the control of glucose homeostasis. We determined that chronic treatment of mice with rapamycin caused hepatic insulin resistance, and using genetic models determined that disruption of mTOR complex 1 (mTORC1), the canonical target of rapamycin, was not the cause of this effect [5]. This surprising result led us to the discovery that not only mTORC1, but also mTOR complex 2 (mTORC2), a second mTOR complex that is not acutely sensitive to rapamycin, was disrupted by chronic rapamycin treatment in tissues including liver, white adipose tissue, and skeletal muscle. Genetic deletion of *Rictor*, an essential protein subunit of mTORC2, in either the whole body of an adult mouse or specifically in the liver mimicked the effects of rapamycin, producing glucose intolerance and hepatic insulin resistance.

Prior to our discovery, the prevailing hypothesis in the field was that the pro-longevity effects of rapamycin were a result of the inhibition of mTORC1 signaling, mediated by substrates including S6K1, 4E-BP1, and ULK1. Backing this theory was work from many labs demonstrating that mice lacking *S6K1^−/−^*, as well as yeast and *C. elegans* with reduced levels of ribosomal subunits and translation initiation factors, have extended longevity (reviewed in [2]). However, our finding that rapamycin also inhibited mTORC2 opened the door to the possibility that decreased mTORC2 signaling might also mediate some of the beneficial effects of rapamycin. To assess this possibility, we examined the longevity of three genetic mouse models of decreased mTORC2 signaling – mice heterozygous for *Rictor*, mice lacking hepatic *Rictor*, and mice in which *Rictor* was deleted from the whole body of mice at 10 weeks or 9 months of age using a tamoxifen-responsive Cre recombinase.

**Figure 1:**
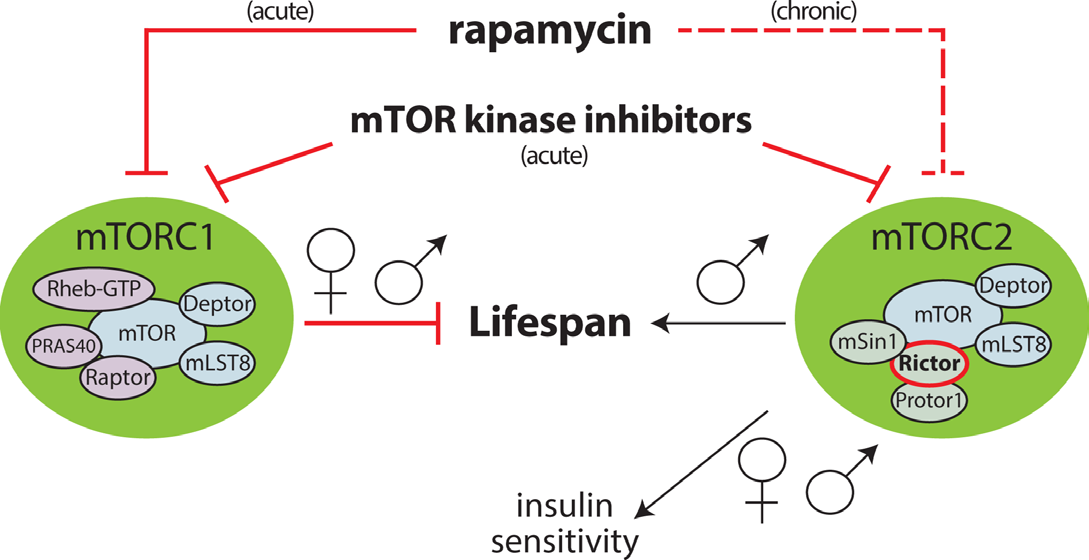
Regulation of lifespan by mTOR The activity of mTORC1 normally acts to limit lifespan, most likely via the promotion of protein translation and the suppression of autophagy (reviewed in [2]). We have determined that mTORC2 is required for the normal lifespan of male, but not female, mice [6]. Chronic treatment with rapamycin, which inhibits both mTORC1 and mTORC2, is more beneficial to females than males, which may be due to the negative influence of mTORC2 inhibition on male lifespan. Sex-based side effects may be seen during the clinical use of mTOR kinase inhibitors for cancer treatment.

We demonstrate in our latest work, now published in *Aging Cell*, that the deletion of *Rictor* in all three models is deleterious for the longevity of males, but surprisingly does not negatively impact female lifespan [6]. We initially suspected that the negative effect of *Rictor* deletion might be a result of glucose intolerance, which we had previously observed in mice lacking hepatic *Rictor*, but surprisingly this was independent from the role of *Rictor* in lifespan. While we cannot yet rule out an mTORC2-independent role for *Rictor*, these results suggest that even partial inhibition of mTORC2 signaling, as observed in mice heterozygous for *Rictor*, is likely to impair the health and longevity of males. As outlined in Figure 1, we theorize that the benefits of rapamycin mediated by inhibition of mTORC1 in males are partially counteracted by the negative effects of mTORC2 inhibition. Conversely, in females, inhibition of mTORC2 is not deleterious. This gender-dependent effect of mTORC2 inhibition may therefore account for the sexually dimorphic efficacy of rapamycin treatment on lifespan [4]. These findings may also have significant implications for the cancer field, as mTOR kinase inhibitors, which acutely inhibit both mTOR complexes (Figure 1), are being developed clinically and may have sex-based side effects. It also suggests that, as we have previously postulated [7], drugs or treatment regimens that specifically inhibit mTORC1 may provide a safer and more efficacious route for the treatment of age-related diseases.

Our findings leave open many questions regarding the role of mTORC2 and lifespan. Most significantly, we do not understand the basis for the sexually dimorphic effect of *Rictor* deletion, and understanding this effect should permit the development of mTOR inhibition strategies that are equally effective in both sexes. Further, although it is clear that inhibition of mTORC2 results in premature mortality in males, the actual cause of death in these animals is unknown. Further complicating the picture, it remains possible that partial inhibition of signaling downstream of mTORC2 may even be beneficial in some contexts – it was recently shown that mice heterozygous for the mTORC2 substrate *Akt1* have extended lifespan [8], while in *C. elegans*, the mTORC2 substrate SGK-1 can alternately promote or retard longevity depending upon the cellular and environmental context [9]. A full realization of the therapeutic potential of rapamycin and mTOR pathway inhibition will only be achieved once the impact of mTORC2 signaling on health and longevity is thoroughly understood.
